# Recovery from Tubular Pregnancy by the Artificial Production of Abortion

**Published:** 1842-06

**Authors:** 


*Recovery from Tubular Pregnancy by the artificial production of Abortion.*—A woman who had borne three children began to complain in the month of May, 1834, of pains in the lower part of the abdomen on the right side in the situation of the ovary. She soon felt a small tumor in this region, which, from the size of a citron, in six weeks acquired that of a child's head. The pain stretched up the back, and down the right thigh, and became every day more insufferable, so as, at last, to deprive her of sleep. Fever, constipation, slight jaundice, and general dropsy, were gradually induced. She believed herself to be again in the family-way, both from the menstrual secretion having been stopped for eleven weeks, and from feeling those peculiar sensations she had experienced in her former pregnancies. Professor Ritgen discovered that the lower part of the uterus was somewhat enlarged, that the orifice was directed to the left side, and that a tumor was in close contact with the upper part of the uterus, and was movable. After a careful examination of it, both from the vagina and the rectum, but especially from examining it by means of the stethoscope, and hearing distinctly the *bruit de soufflet*, he arrived at the conclusion that it was a tubular pregnancy. He, therefore, with the view of aborting it in its early stage, together with the use of enemas, prescribed first Glauber's salts, and afterwards pills composed of the ergot of rye and extract of aloes, covered the lower part of the abdomen with cataplasms, and put a seton in the neighborhood. Vomiting was first excited, then followed contractions of the uterus and of the tumor; black blood flowed from the vagina, at first sparingly, but afterwards in larger quantity, and mixed with clots and shreds, resembling portions of the fœtal membranes. By the fourth day after this, the tamor was found to have diminished considerably in size, and the pain was greatly abated; but a year and a half elapsed before it completely disappeared. Blood was discharged from the vagina for nearly nine months. She has not again become pregnant.—*Ed. Med. and Surg. Jour.* from *Gaz. Med De Paris*, May 8, 1841.

